# The Association Between ABO Blood Group and Preeclampsia: A Systematic Review and Meta-Analysis

**DOI:** 10.3389/fcvm.2021.665069

**Published:** 2021-06-21

**Authors:** Ting Li, Yixiao Wang, Lan Wu, Zhonghui Ling, Chanjuan Li, Wei Long, Kaipeng Xie, Hongjuan Ding

**Affiliations:** ^1^Department of Obstetrics and Gynecology, Women's Hospital of Nanjing Medical University, Nanjing Maternity and Child Health Care Hospital, Nanjing, China; ^2^Women's Hospital of Nanjing Medical University, Nanjing Maternity and Child Health Care Hospital, Nanjing Maternal and Child Health Institute, Nanjing, China

**Keywords:** ABO blood group, preeclampsia, pooled odds ratios, confidence intervals, systematic review, meta-analysis

## Abstract

**Objective:** This meta-analysis comprehensively evaluated the association between ABO blood group and the risk of preeclampsia (PE).

**Design:** Systematic review and meta-analysis.

**Data sources:** PubMed, Web of Science, and ScienceDirect databases from their inception to September 23, 2020.

**Methods:** Pooled odds ratios (ORs) with 95% confidence intervals (CIs) were obtained through random-effects and fixed-effects models according to heterogeneity. Meta-regression analysis was applied to explore the source of heterogeneity. We conducted a subgroup analysis by the publication year, study design, state, and Newcastle-Ottawa Scale (NOS) score. In addition, we calculated the rate of each ABO blood group in PE by total pooled effects.

**Results:** A total of 12 articles with 714,153 patients were included in our analysis. Compared with people without PE (control group), the O blood group presented a lower risk of PE (OR 0.95, 95% CI 0.93–0.97). The AB (OR 1.46, 95% CI 1.12–1.91) blood group presented a higher risk. However, the total pooled OR and 95% CI for the A (OR 1.02, 95% CI 0.90–1.16) and B (OR 1.02, 95% CI 0.98–1.05) blood groups were not significant. The funnel plot and linear regression equation showed that there was no publication bias for the O, A, or B blood groups (all *P* > 0.05). However, the funnel plot and linear regression equation for the AB blood group were obviously asymmetric (*P* < 0.05), and the publication bias persisted even after the trim-and-fill method was applied (*P* < 0.05). Multivariable meta-regression analysis did not find a specific source of heterogeneity. The A blood group showed an association with early-onset PE (OR 0.53, 95% CI 0.33–0.83), and the other blood groups showed no significant differences. In PE, the rates of the O, A, B, and AB blood groups decreased gradually (0.39, 0.33, 0.19, 0.07).

**Conclusion:** These findings suggest that pregnant women with AB blood group are more likely to develop PE, and more attention should be paid to AB blood group whose blood pressure is high but not sufficient to diagnose PE.

**Systematic Review Registration:** Prospero CRD42021227930.

## Introduction

Preeclampsia (PE) is a common complication during pregnancy that affects 5–8% of pregnant women (1, 2). PE is associated with a variety of short-term and long-term complications in mothers and infants, such as placental abruption, cardiovascular disease, renal disease, metabolic disorders, fetal growth restriction (FGR), and preterm birth (3–5). After 20 weeks of gestation, women with systolic BP ≥140 mmHg or diastolic BP ≥90 mmHg on two occasions with or without proteinuria and renal, liver, lung, or neurological organ dysfunction were considered to have PE (5, 6). PE can be categorized as early onset (<34 weeks of gestation) or late onset (≥34 weeks of gestation) (7). In addition, PE can be classified as mild or severe PE depending on the severity of the condition (diagnostic criteria for severe PE are shown in Appendix S1 in Supplementary Material) (8, 9). Risk factors associated with the development of PE have been reported, including previous history of PE, history of abnormal blood pressure, history of gestational diabetes mellitus, multiple pregnancy, and nulliparity (10, 11). Additionally compared with pregnant women without PE, mounting evidence suggests that lower placental growth factor (PlGF) and higher soluble fms-like tyrosine kinase 1 (sFlt-1) levels of maternal blood during pregnancy are linked to PE. Thus, these factors and blood biomarkers may be used for risk prediction of PE before the appearance of the clinical syndrome (6, 12, 13).

In 1901, the ABO blood group system was first discovered and defined by Karl Landsteiner in Austria. It includes types A, B, AB, and O, which are defined according to the expression of agglutinins A and B (14). In recent years, an increasing number of studies on ABO blood groups have been conducted, and blood group has been reported to be associated with the development of many human diseases, such as thrombotic vascular diseases (15), gestational diabetes mellitus (GDM) (16), acute respiratory distress syndrome (ARDS) (17), cardiovascular disease (CVD) (18), gastric cancer (19), infectious diseases (20), and PE (21). Although, the ABO system has been studied for more than a century, its clinical biological significance remains ambiguous.

In 2008, one study evaluated the association between ABO blood group and vascular disease and indicated that non-O blood group was at higher risk for some vascular diseases compared with O blood group (21). In 2013, a systematic review and meta-analysis reported that the AB blood group was associated with the occurrence of PE (22). A systematic review from 2016 aiming to elucidate the association of ABO blood groups with pregnancy-related complications indicated that women with a non-O blood group have an increased risk of PE (23). However, the results of subsequent studies have been inconsistent. Two studies found that patients with blood group AB have a higher risk of PE (24, 25), but another three studies considered that there was no distinct association between ABO blood group and PE (26–28). These five studies included four case–control studies and one cross-sectional study. The largest study population was 17,564 individuals, and the smallest study population was 147 individuals. Hence, we conducted this meta-analysis to comprehensively evaluate the association between ABO blood group and the risk of PE. In addition, we calculated the specific rate of each ABO blood group in PE.

## Methods

This systematic review was conducted according to the Preferred Reporting Items for Systematic Reviews and Meta-Analyses (PRISMA) guidelines (29).

### Literature Search

We searched for the relevant literature through the PubMed, Web of Science, and ScienceDirect databases from inception to September 23, 2020. The Population, Intervention, Comparator, Outcomes and Study designs (PICOS) principle was used to identify articles in the various databases (Appendix S2 in Supplementary Material). We restricted the language to English. We also tracked references to relevant articles. The details of the search process are shown in Appendix S3 in Supplementary Material. Two authors independently collected and integrated the data.

### Eligibility Criteria

We selected articles on the basis of the database searches and applied EndNote X9 to remove duplicate articles. Then, we browsed the titles and abstracts to exclude unrelated articles. This meta-analysis followed the following inclusion criteria: (1) included data on ABO blood group for pregnant women; (2) included pregnant women with and without preeclampsia; (3) prospective and retrospective studies. Reviews, meta-analysis, articles lacking relevant data, letters, and abstracts were excluded.

### Data Extraction and Study Quality Assessment

Two authors independently reviewed each study and decided whether it was eligible for inclusion in our meta-analysis, and if there was any disagreement, the corresponding author joined the discussion. We extracted the following data from the articles: first author name, year of publication, study design, state, and conclusions. The extracted data provided sufficient information for the construction of 2 × 2 tables. The study quality assessment was based on the Newcastle–Ottawa Scale (NOS, Australia and Ottawa, Canada) (30). Using this protocol, the maximum score for each study was nine. Studies with a score ≥7 were regarded as high-quality articles (31). Subgroup analysis was based on the publication year (<2010, ≥2010), study design, state, and NOS score (<7, ≥7) to further evaluate the association between ABO blood group and the risk of PE. Meanwhile, we assessed the association of ABO blood group with mild or severe PE and early-onset or late-onset PE. In addition, we calculated the rate of ABO blood group in PE based on the included studies.

### Statistical Analysis

All the data were analyzed *via* R version 3.6. Forest plots were constructed to obtain Pooled Odds Ratios (ORs) and 95% Confidence Intervals (95% CIs). If *I*^2^ < 50% and *P*_*heterogeneity*_ > 0.05, the fixed-effects model was applied to calculate pooled effect estimates. If *I*^2^ ≥ 50% or *P*_*heterogeneity*_ ≤ 0.05, the random-effects model was applied. We conducted leave-one-out sensitivity analysis by removing each study to explore the robustness of the included literature. Publication bias was evaluated by funnel plots and linear regression equations. If the funnel plots were obviously asymmetric, we further adjusted the data by the trim-and-fill method. In addition, the multivariable meta-regression analysis was conducted to explore the source of heterogeneity on the basis of publication year, NOS score, state, and study design. We pooled the rates of O, A, B, and AB blood group in PE as proportions with 95% CIs after log transformation. A cut-off value of *P* < 0.05 was defined as statistically significant.

## Results

### Study Selection

**Figure 1:**
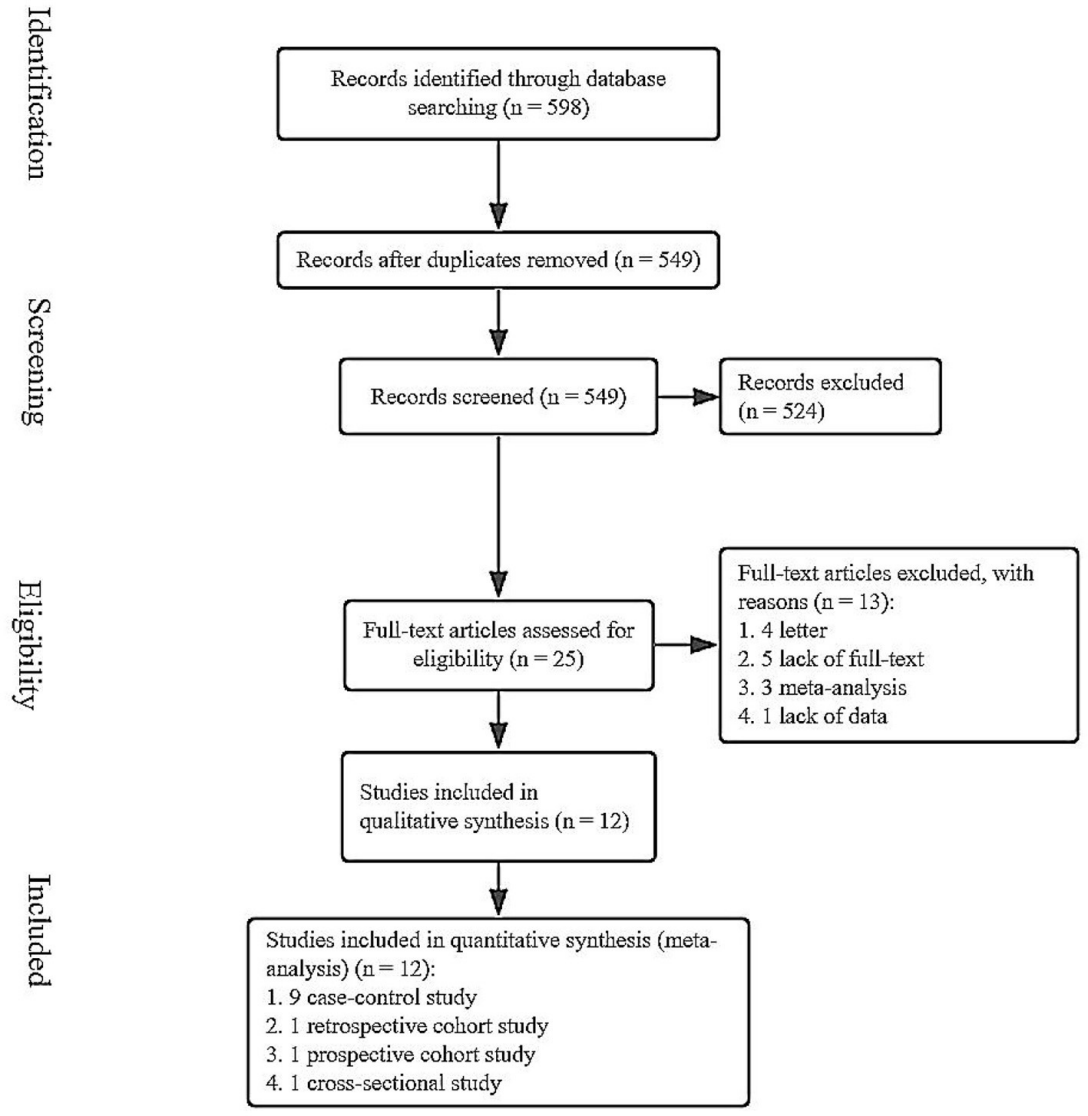
A flow chart of screened studies on ABO blood group and PE.

We searched for the relevant literature through PubMed, Web of Science, and ScienceDirect databases from inception to September 23, 2020. A total of 598 studies were obtained (Figure 1). After removing duplicate articles, 549 articles remained. Then, irrelevant and data-deficient articles were eliminated by browsing the titles, abstracts, and full text. Finally, we included 12 articles in this meta-analysis (24–28, 32–38).

### Study Characteristics

The main characteristics of the included studies are shown in Table 1. This meta-analysis included 12 articles with 714,153 patients: nine case–control studies, one cross-sectional study, one retrospective cohort study, and one prospective cohort study. The publication dates of these articles ranged from 1976 to 2020. Among these articles, the study areas included Europe for four studies; Asia, four; North America, two; South America, one; and Africa, one. The smallest sample size was 90, and the largest sample size was 679,740. The conclusions of seven articles indicated that there was no effect of ABO blood group on the risk of PE. Four studies showed that AB blood group increased the risk of PE. One study indicated that non-O blood groups had significantly higher odds of PE. One study showed that the A blood group increased the risk of PE.

**Table 1:** Characteristics of the included articles.

**First author**	**Year**	**Country (state)**	**Study design**	**N**	PE: Control^*^	**O (PE: control)**	**A (PE: control)**	**B (PE: control)**	**AB (PE: control)**	**Conclusions**
Okoye HC	2020	Nigeria (Africa)	Cross-sectional	147	66: 81	46: 49	12: –	7: –	1: –	No effect of ABO blood types on the risk of PE
Mahasub N	2020	Thailand (Asia)	Case–control	690	230: 460	72: 164	53: 99	88: 171	17: 26	No effect of ABO blood types on the risk of PE
Burgess A	2019	Pennsylvania (North America)	Case–control	511	252: 259	106: 122	95: 100	38: 32	13: 5	AB blood group increased the risk of PE
Aghasadeghi F	2017	Iran (Asia)	Case–control	331	121: 210	61: 93	28: 63	26: 46	6: 8	No effect of ABO blood types on the risk of PE
Avci D	2016	Turkey (Asia)	Case–control	17,564	250: 17,314	69: 5,423	104: 7,756	46: 2,819	31: 1,316	AB blood group increased the risk of PE
Phaloprakarn C	2013	Thailand (Asia)	Case–control	5,320	350: 4,970	105: 1,851	100: 1,053	113: 1,719	32: 347	A and AB blood group increased the risk of PE
Lee BK	2012	Sweden (Europe)	Retrospective cohort	679,740	37,814: 641,926	13,881: 243,041	17,408: 291,453	4,430: 74,147	2,095: 33,285	Non-O blood group increased the risk of PE
Alpoim PN	2011	Brazil (South America)	Case–control	90	55: 35	17: 15	–	–	–	No effect of ABO blood types on the risk of PE
Hiltunen LM	2009	Finland (Europe)	Case–control	927	248: 679	72: 217	104: 294	40: 124	32: 44	AB blood group increased the risk of PE
Clark P	2008	Scotland (Europe)	Prospective cohort	3,985	66: 3,919	32: 2,055	–	–	–	No effect of ABO blood types on the risk of PE
Witsenburg C P	2005	Netherlands (Europe)	Case–control	308	36: 272	11: 111	–	–	–	No effect of ABO blood types on the risk of PE
Scott JR	1976	Iowa (North America)	Case–control	4,540	46: 4,494	22: 2,139	15: 1,705	7: 511	2: 139	No effect of ABO blood types on the risk of PE

* *Control means pregnant women without preeclampsia (PE). –, No data are available in the article*.

### Total Pooled Effect

**Figure 2:**
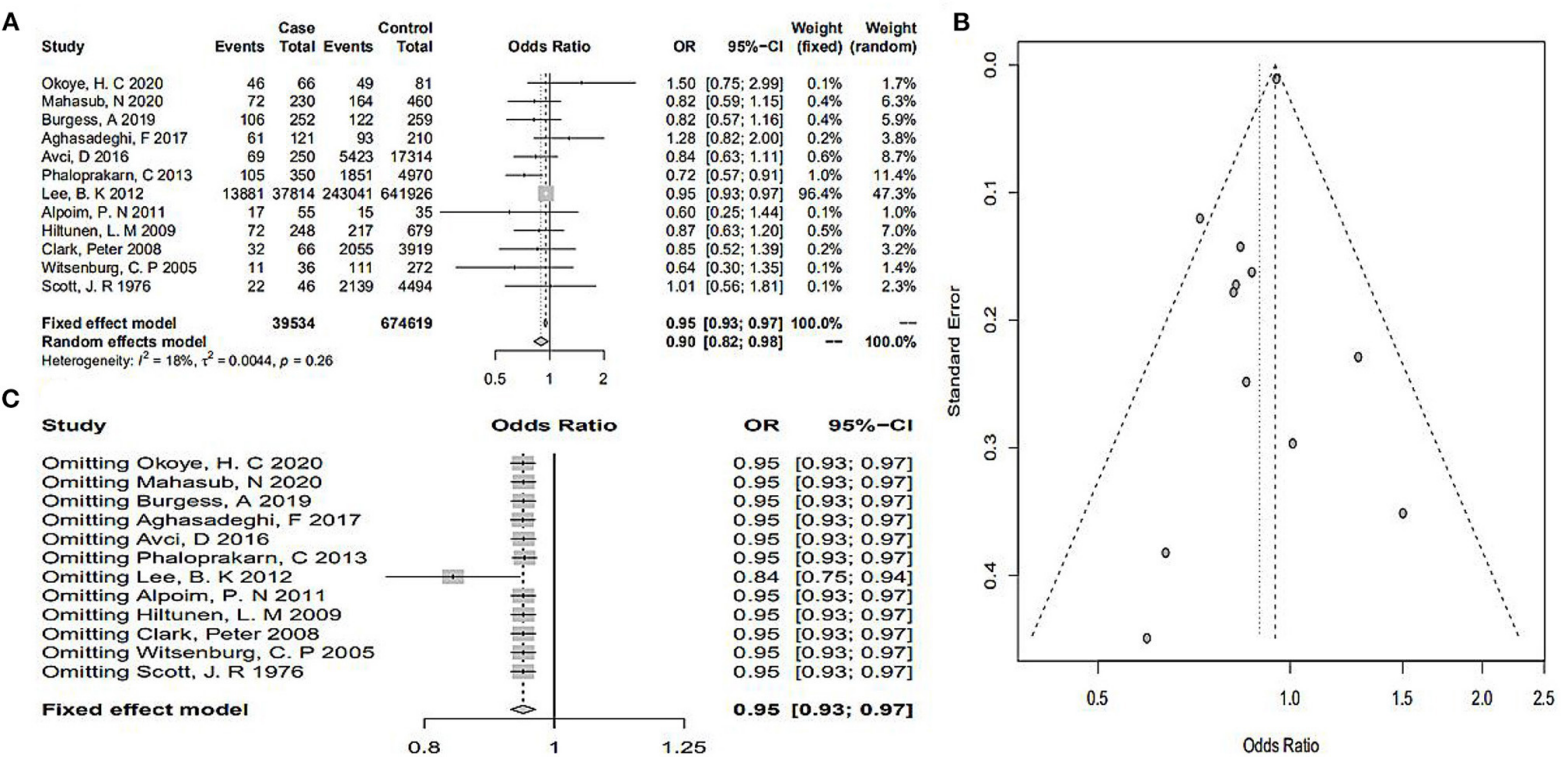
**(A)** Forest plots of the risk of PE in the O blood group. **(B)** Funnel plot of the included studies for the O blood group. **(C)** Sensitivity analysis of the included studies for the O blood group.

**Figure 3:**
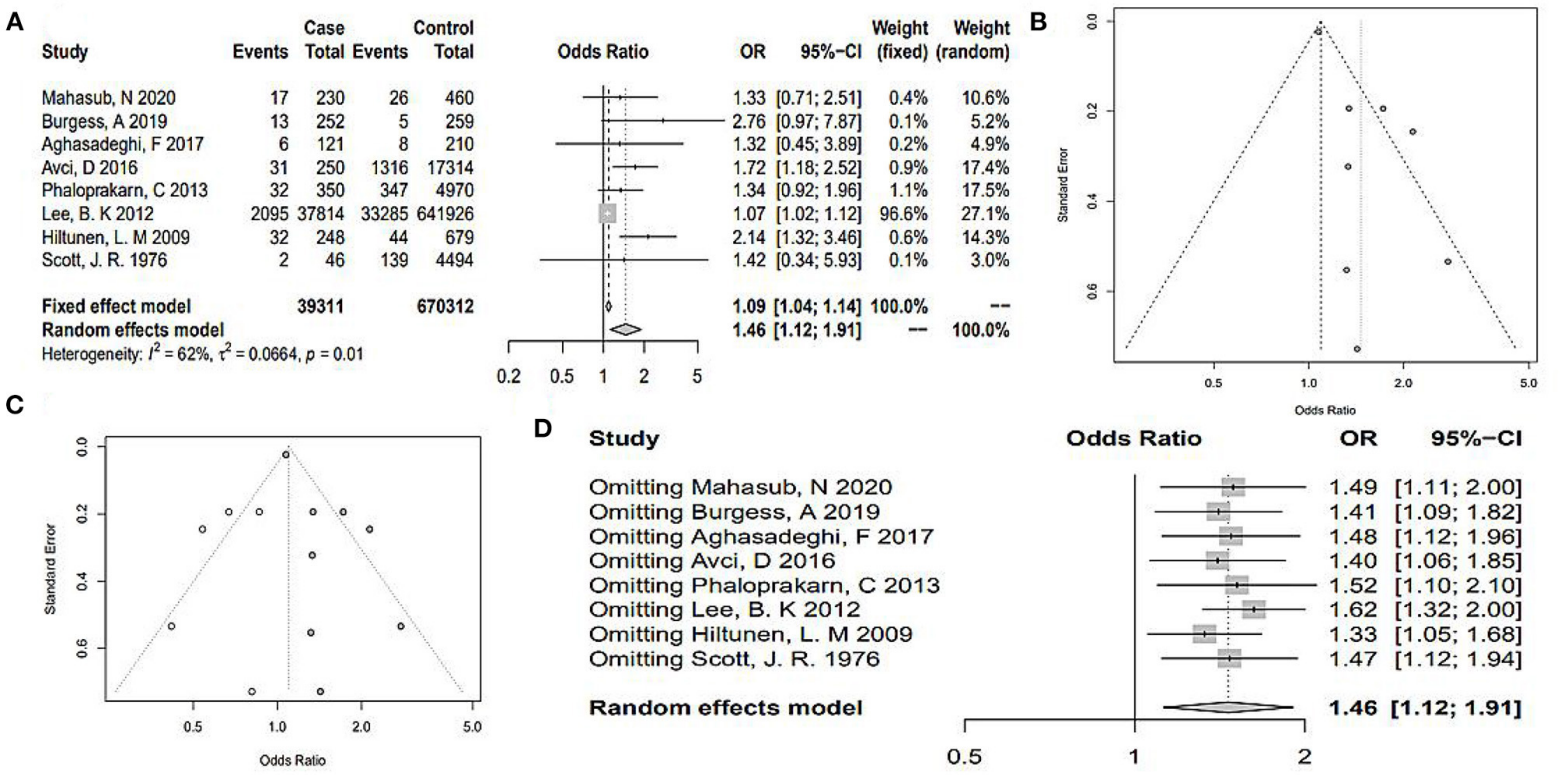
**(A)** Forest plots of the risk of PE in the AB blood group. **(B)** Funnel plot of the included studies for the AB blood group. **(C)** Funnel plot of the included studies for the AB blood group after trim-and-fill method. **(D)** Sensitivity analysis of the included studies for the AB blood group.

**Figure 4:**
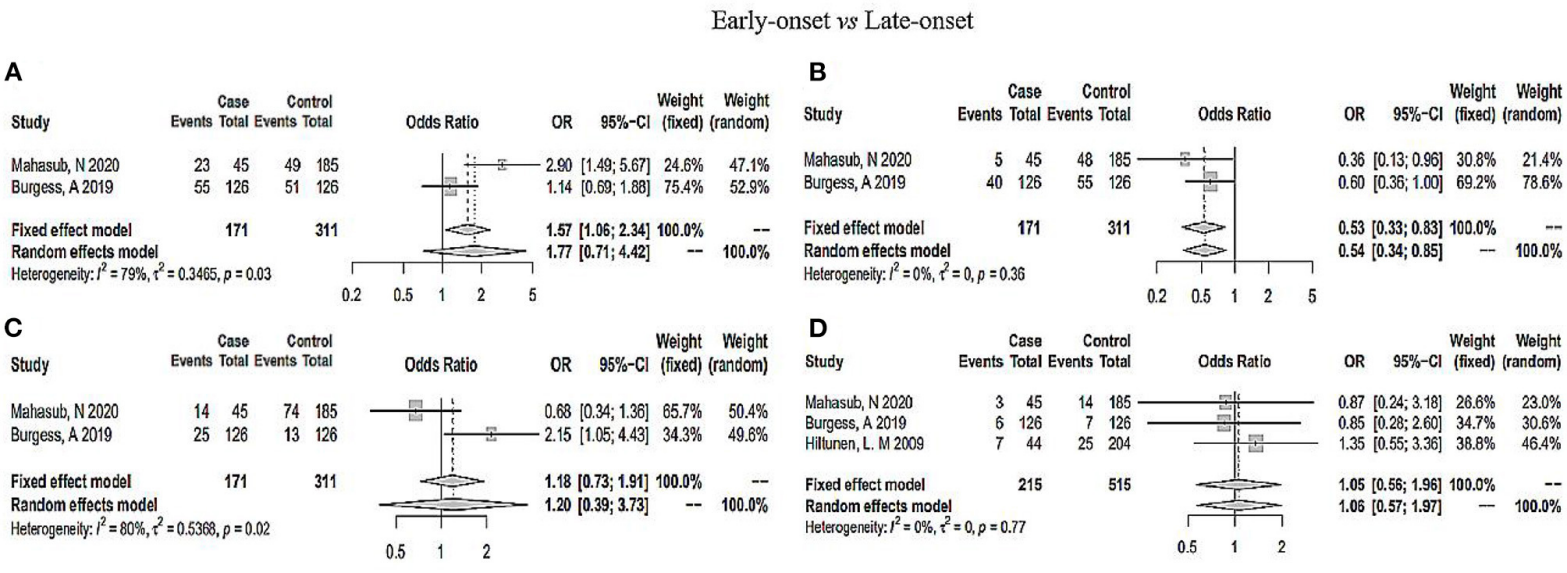
Analysis of early-onset (case) and late-onset (control) PE in ABO blood group. **(A)** O blood group; **(B)** A blood group; **(C)** B blood group; **(D)** AB blood group.

As shown in Figure 2A, the heterogeneity among the eligible articles was *I*^2^ = 18% (*P* = 0.26), so we chose a fixed-effects model. The total pooled effect showed that the O blood group presented as a protective factor against PE (OR 0.95, 95% CI 0.93–0.97). At the same time, we calculated the outcomes for A, B, and AB blood groups. The AB blood group presented a high risk of PE in the random-effects model, respectively (OR 1.46, 95% CI 1.12–1.91, *I*^2^ = 62%, *P*_*heterogeneity*_ = 0.01, Figure 3A). However, the total pooled OR and 95% CI showed no significance of the A blood group in the random-effects model (OR_A_ 1.02, 95% CI_A_ 0.90–1.16, $I_{\text{A}}^{2}$ = 49%, *P*_*heterogeneity*_ = 0.05) and B blood group in the fixed-effect model (OR 1.02, 95% CI 0.98–1.05, *I*^2^ = 0%, *P*_*heterogeneity*_ = 0.81) (Figures S1A, S2A). Although, only some articles studied mild or severe PE and early-onset or late-onset PE, we analyzed them further. As Figure S3 shows, regardless of mild or severe PE, there was no association between the ABO blood group and PE. However, the A blood group showed an association with early-onset PE, and the other blood groups showed no significance (OR 0.53, 95% CI 0.33–0.83, *I*^2^ = 0%, Figure 4).

### Publication Bias and Sensitivity Analysis

The funnel plot and linear regression equation showed that there was no publication bias with respect to the effects of the O, A, and B blood group (*P*_O_ = 0.59, *P*_A_ = 0.67, *P*_B_ = 0.90) (Figure 2B, Figures S1B, S2B). The funnel plot of AB blood group was clearly asymmetrical (Figure 3B). We further conducted the trim-and-fill method; the funnel plot was symmetric, but publication bias still existed (*P* < 0.05, Figure 3C). As Figure 2C shows, when omitting one of these studies (28), the sensitivity analysis of the O blood group showed an OR of 0.95 (95% CI 0.93–0.97), nearly the same outcome as the total pooled effect (OR 0.95, 95% CI 0.93–0.97). Similarly, when omitting any one of the other studies, the outcomes showed that O blood group was a protective factor against PE. The sensitivity analysis of the AB blood group showed similar outcomes after omitting any one study (Figure 3D). The sensitivity analysis of the A and B blood groups showed that after omitting any one study, the effect of the A and B blood groups was not significant (Figures S1C, S2C).

### Multivariable Meta-Regression Analysis

In the total pooled effect, heterogeneity of the AB blood group was *I*^2^ = 62.0% (*P* < 0.05, Figure 3A). Thus, we conducted multivariable meta-regression analysis on the basis of publication year, NOS score, state, and study design. The results confirmed that these factors showed no significant effect on the heterogeneity (all *P* > 0.05, Table 2).

**Table 2:** Results of meta-regression analysis.

**Study-level variables**	**Coefficient (95% CI)**	***P*-value**
Publication year	(−0.03, 0.05)	0.6
NOS score	(−0.59, 0.30)	0.53
State	(−0.26, 0.91)	0.28
Study design	(−31.91, 14.40)	0.46

*NOS, Newcastle–Ottawa Scale*.

### Subgroup Analysis

Subgroup analysis was based on the study design, state, NOS score (<7, ≥7) and publication year (<2010, ≥2010) to further evaluate the association between ABO blood group and the risk of PE. As shown in Table 3, the outcomes in the O blood group were almost the same. When publication year <2010 and studies performed in America, the outcomes showed no significance (all *P* > 0.05). Subgroup analysis of cross-sectional study design and studies performed in Africa, which included only one study each, showed no significance. The outcome for European region of AB blood group was not significant for PE (N_Europe_ = 2, OR_Europe_ 1.45, 95% CI_Europe_ 0.74–2.84) (Table 4). The subgroup analysis of the A blood group was somewhat inconsistent and showed no significance (Table S1). Only the outcomes of cohort study, NOS score ≥ 7 and European region showed significant differences (N_cohort study_ = 1, OR_cohort study_ 1.03, 95% CI_cohort study_ 1.00–1.05; N_Europe_ = 2, OR_Europe_ 1.03, 95% CI_Europe_ 1.01–1.05; N_NOS score ≥7_ = 3, OR_NOS score ≥7_ 1.03, 95% CI_NOS score ≥7_ 1.01–1.05). The subgroup analysis of the B blood group was almost consistent (Table S2).

**Table 3:** Subgroup analysis of the risk of PE in O blood group.

**Subgroup**	**Studies (*N*)**	**PE: control**	**O (PE: control)**	***I*^**2**^**	**OR (95% CI)**
**Study design**					
Case–control study	9	1,588: 28,693	535: 10,135	0%	0.82 (0.73–0.93)
Cohort study	2	37,880: 645,845	13,913: 245,096	0%	0.95 (0.93–0.97)
Cross-sectional study	1	66: 81	46: 49	–	1.50 (0.75–2.99)
**State**					
Asia	4	951: 22,954	307: 7,531	39%	0.82 (0.71–0.95)
Europe	4	38,164: 646,796	13,996: 245,424	0%	0.95 (0.93–0.97)
America	3	353: 4,788	145: 2,276	0%	0.83 (0.63–1.10)
Africa	1	66: 81	46: 49	–	1.50 (0.75–2.99)
**NOS score**					
<7	8	1,176: 31,473	423: 11,809	0%	0.82 (0.72–0.94)
≥7	4	38,358: 643,146	14,071: 243,471	0%	0.95 (0.93–0.97)
**Publication year**					
<2010	4	396: 9,364	137: 4,522	0	0.86 (0.68–1.08)
≥2010	8	39,138: 665,255	14,357: 250,758	41%	0.95 (0.93–0.97)

*NOS, Newcastle–Ottawa Scale; PE, preeclampsia*.

**Table 4:** Subgroup analysis of the risk of PE in AB blood group.

**Subgroup**	**Studies (*N*)**	**PE: control**	**AB (PE: control)**	***I*^**2**^**	**OR (95% CI)**
**Study design**					
Case–control study	7	1,497: 28,386	133: 1,885	0%	1.63 (1.32–2.00)
Cohort study	1	37,814: 641,926	2,095: 33,285	–	1.07 (1.02–1.12)
**State**					
Asia	4	951: 22,954	86: 1,697	0%	1.47 (1.15–1.87)
Europe	2	38,062: 642,605	2,127: 33,329	87%	1.45 (0.74–2.84)
America	2	298: 4,753	15: 144	0%	2.27 (1.01–5.13)
**NOS score**					
<7	5	1,019: 27,247	2,144: 33,355	0%	1.07 (1.03–1.12)
≥7	3	38,292: 643,065	84: 1,815	0%	1.68 (1.34–2.09)
**Publication year**					
<2010	2	294: 5,173	34: 183	0%	2.06 (1.31–3.23)
≥2010	6	39,017: 665,139	2,194: 34,987	53%	1.34 (1.04–1.72)

*NOS, Newcastle–Ottawa Scale; PE, preeclampsia*.

### Rates of ABO Blood Group in PE

**Figure 5:**
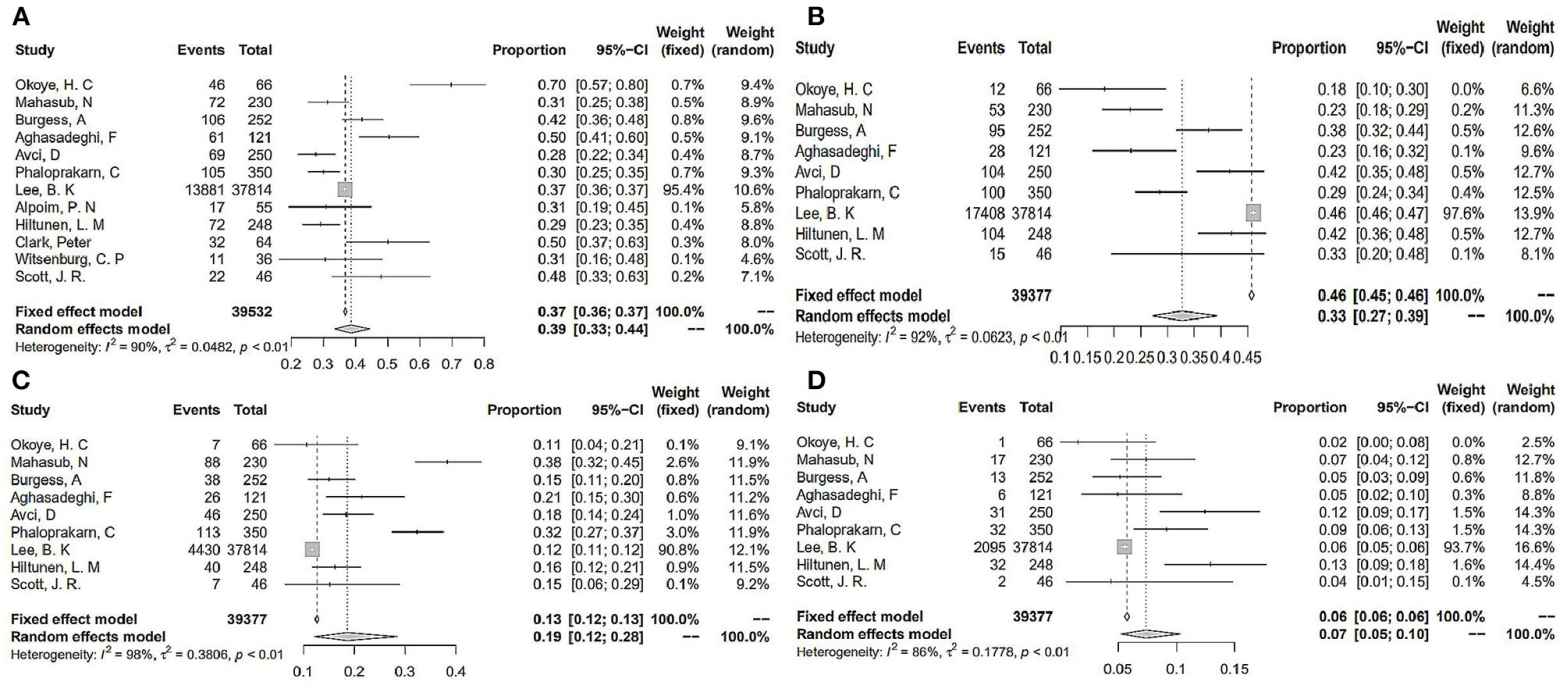
The rates of ABO blood group in PE. **(A)** O blood group; **(B)** A blood group; **(C)** B blood group; **(D)** AB blood group.

The rates of the O, A, B, and AB blood groups were further analyzed by forest plots. As shown in Figure 5, we can see that the rate of the O blood group was 0.39 (95% CI 0.33–0.44, *I*^2^ = 90%). In PE, the rates of the A, B, and AB blood group decreased gradually (0.33, 0.19, and 0.07).

## Discussion

Our systematic review and meta-analysis comprehensively explored the association between ABO blood group and PE. Twelve articles comprising 714,153 patients were included. On the basis of previous studies and the outcomes we obtained, the present study demonstrated that compared with the control group, the O blood group presented as a protective factor for PE. Conversely, the AB blood group aggravated the risk of PE, and the A and B blood groups showed no significant effect on the risk of PE. Notably, we found that the A blood group showed an association with early-onset PE. In addition, we further calculated the specific incidences of the ABO blood groups in PE. The rate of the O blood group in PE was 0.39 (95% CI 0.33–0.44, *I*^2^ = 90%), and the rates of the A, B, and AB blood groups were 0.33, 0.19, and 0.07, respectively.

ABO blood group antigens exist on many kinds of cells of the human body; in addition to common red blood cells, these antigens are also expressed on vascular endothelial cells and neuronal cells (39). Existing studies found that ABO blood group status is correlated with many diseases, such as CVD, ARDS, GDM, and PE (16–18, 27, 28). Nevertheless, the association between PE and ABO blood group has been controversial. In 1976, an article published in *JAMA* studied 23 patients with PE, 23 patients with eclampsia, and 4,494 controls and suggested that there was no association between ABO blood group and PE (controls vs. PE: O blood group, 47.6 vs. 47.8%; A blood group, 37.9 vs. 32.6%; B blood group, 11.4 vs. 15.2%; AB blood group, 3.1 vs. 4.3%; all *P* > 0.05) (32). Studies performed in 2005 and 2008 arrived at the same conclusion. A meta-analysis also concluded that non-O blood groups are more susceptible to certain vascular diseases than O blood group (21). In 2009, for the first time, a population-based nested case–control study indicated that AB blood group increased the risk of PE (controls vs. PE: O blood group, 32.0 vs. 29.0%, *P* = 0.4; A blood group, 43.3 vs. 41.9%, *P* = 0.7; B blood group, 18.3 vs. 16.1%, *P* = 0.5; AB blood group, 6.5 vs. 12.9%, *P* = 0.002) (35). This outcome was consistent with ours. Subsequently, Alpoim et al. studied the association between severe PE (sPE) and ABO and indicated that there was no effect of ABO blood group on the risk of PE. In 2012, the team of Lee conducted a cohort study of 641,926 pregnant women and used two models for systematic analysis. After adjusting model 1 (age, country of origin, calendar year, smoking, and RhD status), women in blood group AB had an increased risk of PE (OR 1.10, 95% CI 1.04–1.16, *P* < 0.001) and an even higher increase in risk for sPE (OR 1.18, 95% CI 1.07–1.30, *P* < 0.001). The same outcomes were also obtained in the A and B blood groups. Similar outcomes were obtained from model 2, which adjusted for the model 1 covariates and for BMI, diabetes, and hypertension. For example, women in the AB blood group had an increased risk of PE and sPE (OR 1.12, 95% CI 1.05–1.21, *P* < 0.001; OR 1.20, 95% CI 1.08–1.35, *P* < 0.001). In summary, the outcomes showed that patients with non-O blood groups had an increased risk of PE (37). Furthermore, in 2013, a systematic review and meta-analysis that included only two eligible articles reported that the AB blood group was associated with the occurrence of PE (OR 2.42, 95% CI 1.63–3.58, *P* < 0.0001) (22). However, the results of subsequent studies are also inconsistent. A systematic review from 2016 aimed to elucidate the association of ABO blood groups with pregnancy-related complications, and the results indicated that women with A or AB blood group had an increased risk of PE (O blood group, OR 0.77, 95% CI 0.67–0.88; A blood group, OR 1.78, 95% CI 1.04–3.07; AB blood group, OR 1.94, 95% CI 1.20–3.13) (23). However, the results of subsequent studies have been inconsistent. Two studies found that patients with blood group AB have a higher risk for PE (24, 25), but another three studies considered that there was no distinct association between ABO blood group and PE (26–28). The conclusion of our analysis indicated that the O blood group is a protective factor against PE. Conversely, the blood group AB aggravated the risk of PE, while the A and B blood groups showed no significant effect on the risk of PE. Notably, we found that the A blood group showed an association with early-onset PE. In addition, we further calculated the specific rate of each ABO blood group in PE, and the rates of the O, A, B, and AB blood groups decreased gradually (0.39, 0.33, 0.19, 0.07).

The strengths of our study include that different blood groups were analyzed and that subgroup analysis was carried out in detail. Furthermore, we evaluated both mild and severe PE and both early-onset and late-onset PE. Both funnel plots and linear regression equations were used to calculate publication bias. Multivariable meta-regression analysis on the basis of subgroups was also conducted to explore the source of heterogeneity. In addition, the rates of O, non-O, A, B, and AB blood groups were further specifically analyzed though total pooled effects. Undoubtedly, there are also some limitations in this study. First, only 12 articles were included, and the limited number of studies may influence the outcomes. We restricted the language of studies to English. In addition, in the meta-analysis of 2016, we were unable to find the full text of all included studies. PE is a complex physiological and pathological process, and many factors will affect the occurrence and development of PE (e.g., genetic factors, diet, and environment). It is obviously insufficient to use only the ABO blood group as a factor to predict PE, and all the potential risk factors may act as confounding factors in research outcomes (40–44). Although, the relationship between the ABO blood group system and disease has been studied for a long time, the mechanism of how the ABO blood group system causes and affects disease is not clear. Studies have found that placental protein 13 produced by pregnant women may be associated with the onset of PE by binding to β-galactosides (such as *N*-acetylgalactosamine, galactose, and fucose) at the end of ABO blood group antigens. However, the study on placental protein 13 is not conclusive, but a potential possibility. ABO blood group system has a high degree of polymorphism and it is difficult to simulate ABO blood group antigen in animal models, making it difficult to explore the relationship between ABO blood group and the pathogenesis of PE (45, 46). With the development of molecular biology techniques, transgenic techniques, and bioanalytical tools, we expect to discover how the ABO blood group system causes and affects disease.

## Conclusion

In conclusion, the O blood group is a protective factor against PE. Conversely, the AB blood group aggravates the risk of PE, and the A and B blood groups have no significant effect on the risk of PE. In addition, the A blood group showed an association with early-onset PE. These findings suggested that clinicians should pay more attention to pregnant women with blood group AB whose blood pressure is high but not sufficient to diagnose PE.

## Data Availability Statement

The original contributions presented in the study are included in the article/Supplementary Material, further inquiries can be directed to the corresponding author/s.

## Author Contributions

TL and YW: study design, data extration, statistical analysis, and manuscript writing. LW, ZL, CL, and WL: study design, data extraction, and verification. KX and HD: study design, statistical analysis, manuscript editing and reviewing, and funding. All authors contributed to the article and approved the submitted version.

## Conflict of Interest

The authors declare that the research was conducted in the absence of any commercial or financial relationships that could be construed as a potential conflict of interest.
