# Supplementary material for: The Association Between ABO Blood Group and Preeclampsia: A Systematic Review and Meta-Analysis
Source: Front Cardiovasc Med. 2021 Jun 21;8:665069. doi: 10.3389/fcvm.2021.665069 (PMC8256995; doi:10.3389/fcvm.2021.665069)
Supplement: Supplementary file 1 [file Data_Sheet_1.pdf]

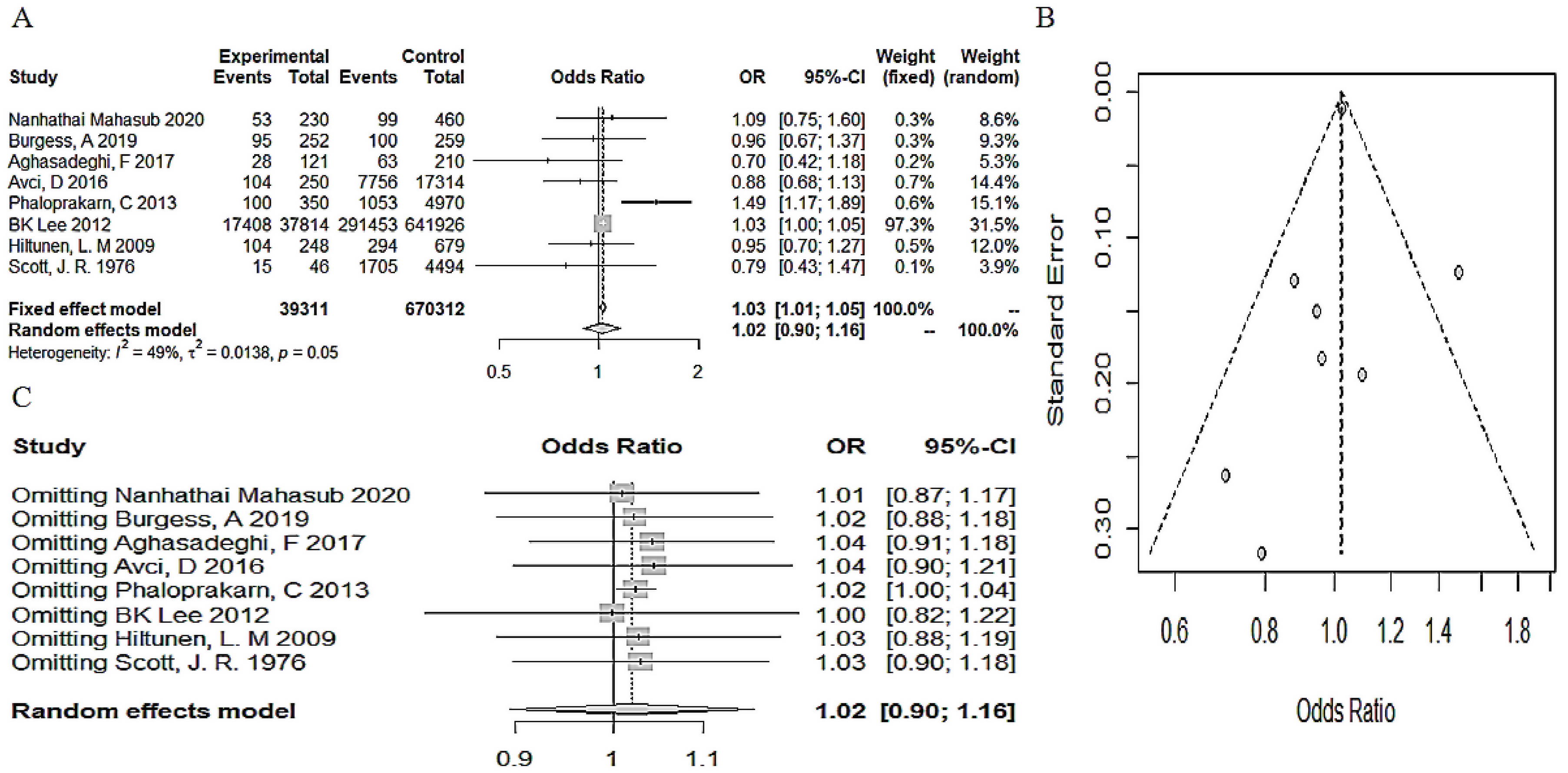

Figure S1. A. Forest plots of the risk of PE in the A blood group; **B.** Funnel plot of the included studies for the A blood group; **C.** Sensitivity analysis of the included studies for the A blood group.

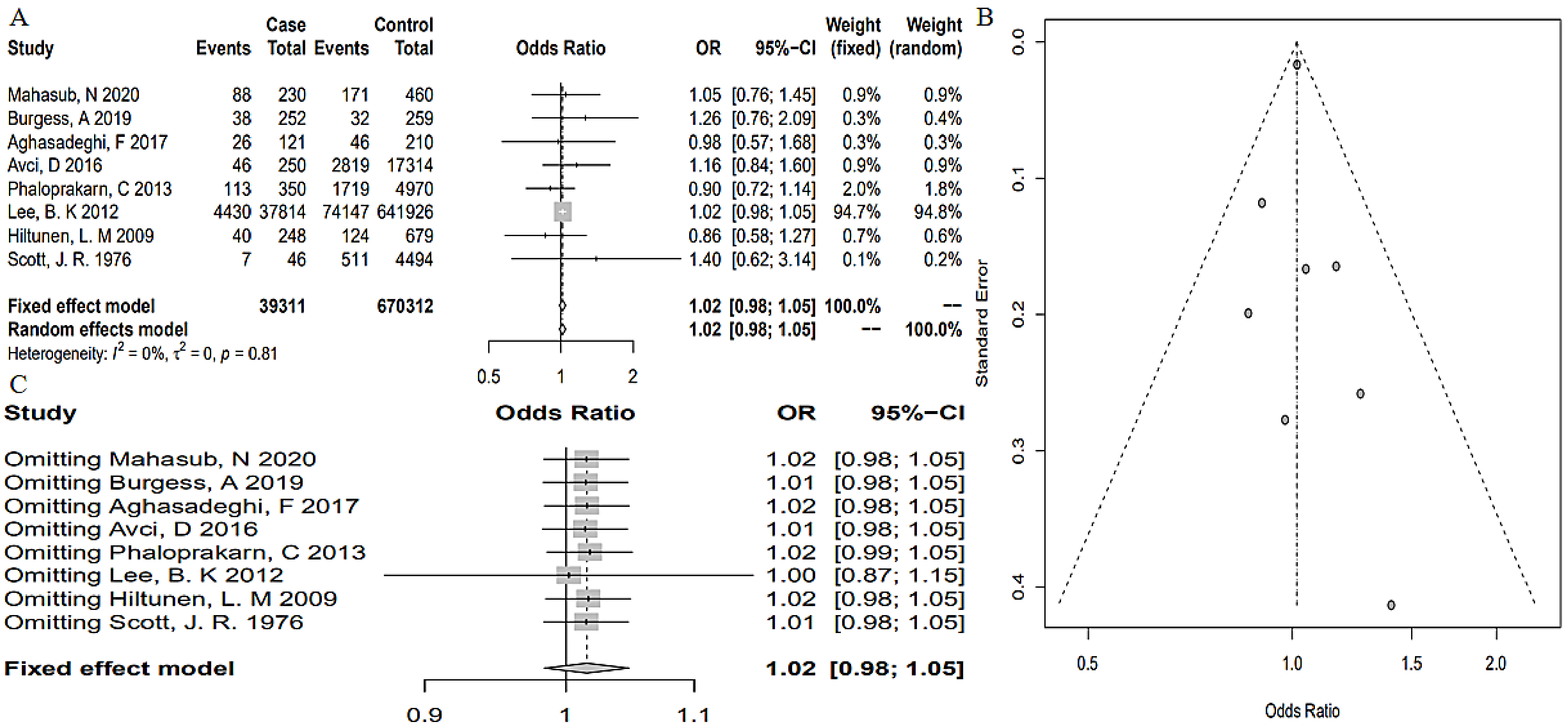

Figure S2. A. Forest plots of the risk of PE in the B blood group; **B.** Funnel plot of the included studies for the B blood group; **C.** Sensitivity analysis of the included studies for the B blood group.

Mild *vs* Severe

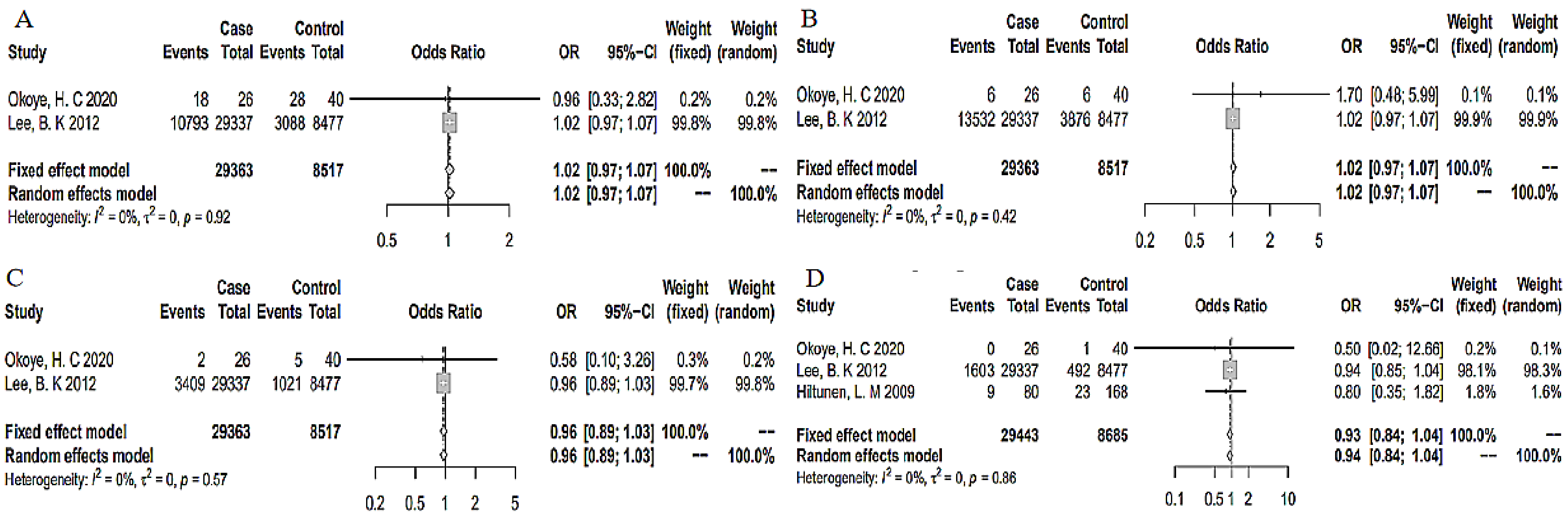

Figure S3. Subgroup analysis of mild and severe PE in ABO blood group. **A**, O blood group; **B**, A blood group; **C**, B blood group; **D**, AB blood group.

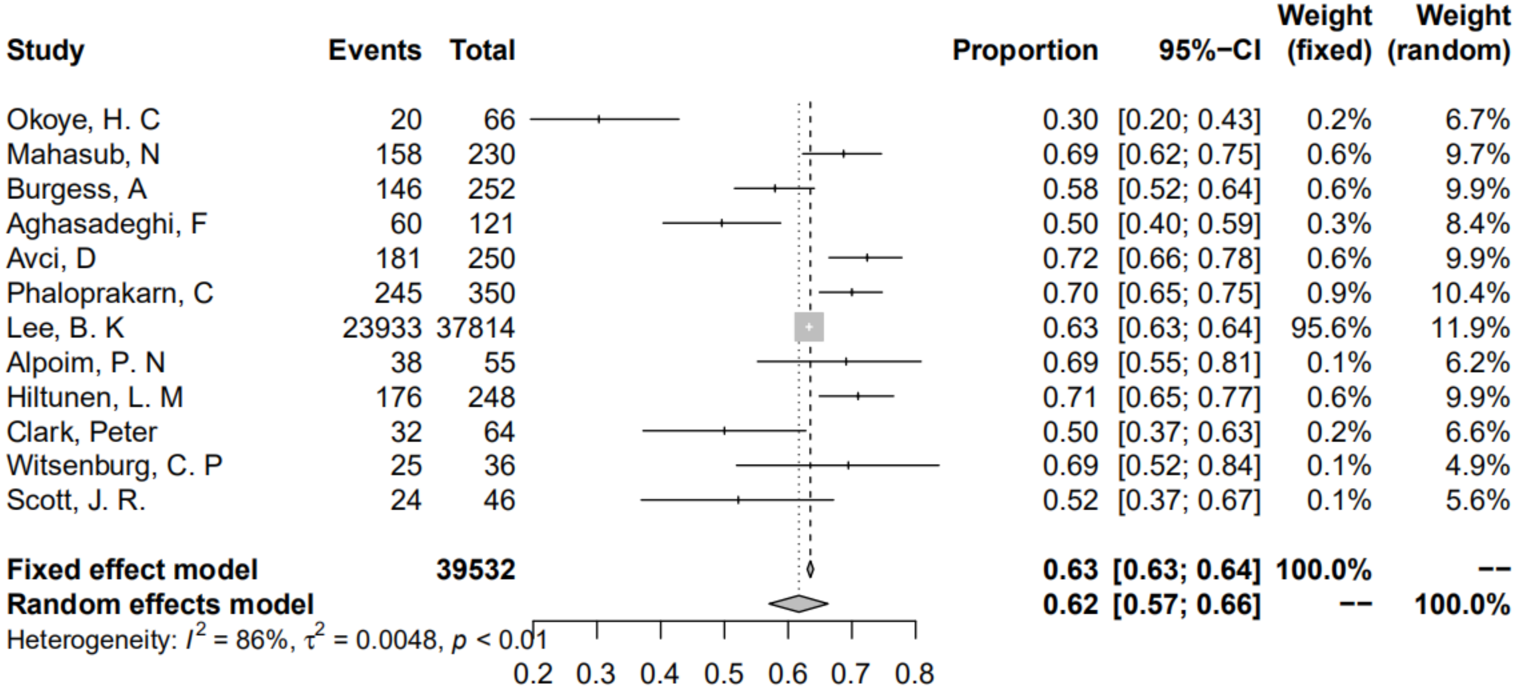

Figure S4. The rate of non-O blood group in PE.

Table S1. Subgroup analysis of the risk of PE in A blood group

Subgroup	Studies (N)	PE : Control	A (PE : Control)	I^2	*OR* (95% CI)
Study design					
Case-control study	7	1497 : 28386	499 : 11070	57%	1.00 (0.82-1.22)
Cohort study	1	37814 : 641926	17408 : 291453	-	1.03 (1.00-1.05)
State					
Asia	4	951 : 22954	285 : 8971	75%	1.03 (0.75-1.43)
Europe	2	38062 : 642605	17512 : 291747	0%	1.03 (1.00-1.05)
America	2	298 : 4753	110 : 1805	0%	0.92 (0.67-1.25)
NOS score					
< 7	5	1019 : 27247	342 : 10677	70%	0.98 (0.73-1.30)
≥ 7	3	38292 : 643065	17565 : 291846	0%	1.03 (1.00-1.05)
Publication year					
< 2010	2	294 : 5173	119 : 1999	0%	0.91 (0.70-1.19)
≥ 2010	6	39017 : 665139	17788 : 300524	61%	1.04 (0.89-1.22)

Table S2. Subgroup analysis of the risk of PE in B blood group

Subgroup	Studies (N)	PE : Control	B (PE : Control)	I^2	*OR* (95% CI)
Study design					
Case-control study	7	1497 : 28386	358 : 5422	0%	1.00 (0.87-1.15)
Cohort study	1	37814 : 641926	4430 : 74147	-	1.02 (0.98-1.05)
State					
Asia	4	951 : 22954	273 : 4755	0%	0.99 (0.85-1.16)
Europe	2	38062 : 642605	4470 : 74271	0%	1.01 (0.98-1.05)
America	2	298 : 4753	45 : 543	0%	1.29 (0.84-1.99)
NOS score					
< 7	5	1019 : 27247	4558 : 74442	0%	0.98 (0.84-1.15)
≥ 7	3	38292 : 643065	230 : 5127	0%	1.02 (0.98-1.05)
Publication year					
< 2010	2	294 : 5173	47 : 635	11%	0.93 (0.66-1.33)
≥ 2010	6	39017 : 665139	4741 : 78934	0%	1.02 (0.98-1.05)
