# Supplementary material for: The Association Between ABO Blood Group and Preeclampsia: A Systematic Review and Meta-Analysis
Source: Front Cardiovasc Med. 2021 Jun 21;8:665069. doi: 10.3389/fcvm.2021.665069 (PMC8256995; doi:10.3389/fcvm.2021.665069)

Appendix S1:

Diagnostic criteria for severe PE:

1. Systolic BP > 160 mmHg or diastolic BP > 110 mmHg on two occasions at least 4 hours apart.

2. Platelet count ＜ $100*10^9$/L.

3. Impaired liver function (Elevated concentrations of blood liver transaminases to twice normal levels and/or severe persistent epigastric pain).

4. Renal insufficiency (Serum creatinine > 1.1 mg/dl or doubling of serum creatinine in the absence of renal disease).

5. New-onset brain or visual disturbance.

6. Pulmonary edema.

Appendix S2:

Search method: Population, Intervention, Control, Outcome, Study Design (PICOS) inclusion criteria.

**Population**	Pregnant women with PE
**Intervention**	Different ABO blood groups
**Control**	Healthy pregnant women or pregnant women without PE
**Outcomes**	The association of different ABO blood groups with PE
**Study design**	Case-control, cohort, cross-sectional studies

Appendix S3:

Search method:

1. Pubmed

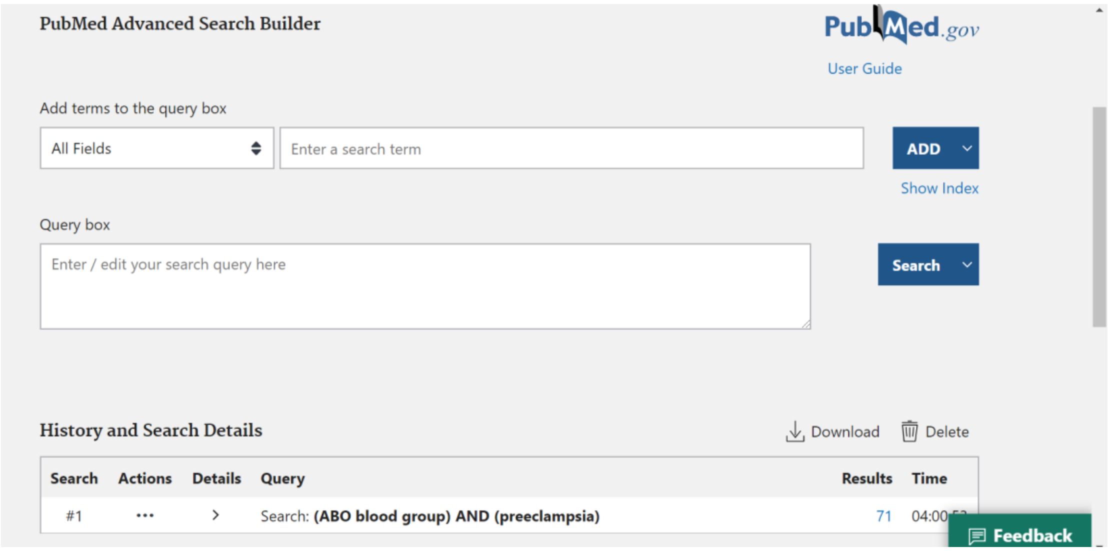

2. Web of science

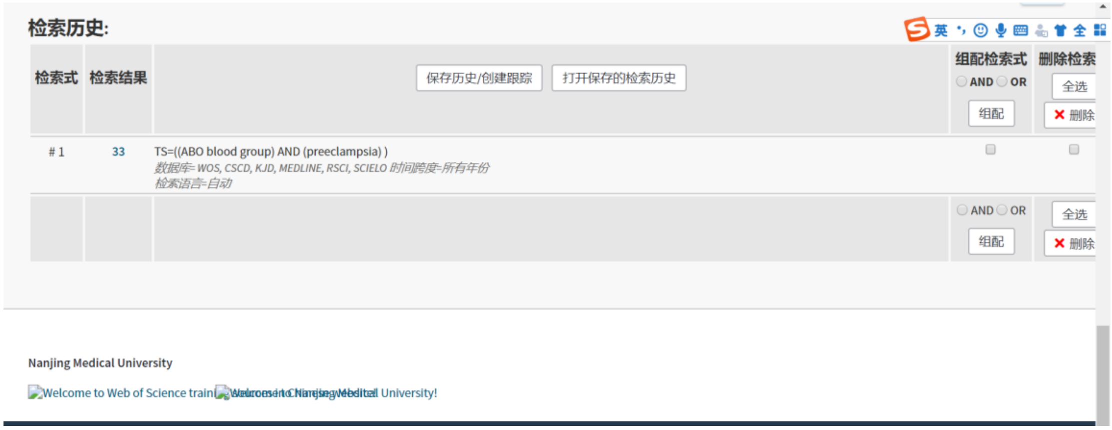

3. ScienceDirect

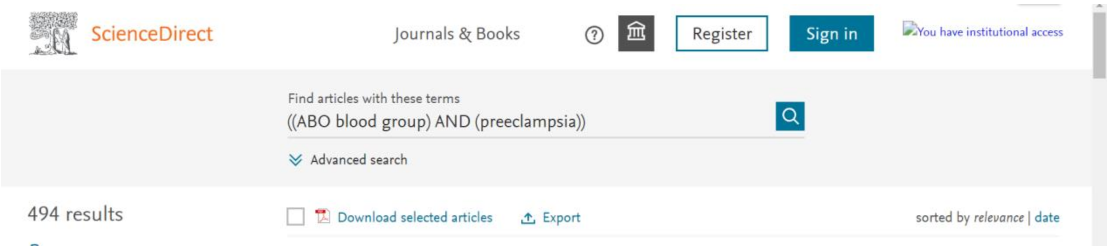

Supplement: Supplementary file 2 [file Data_Sheet_2.pdf]
